# Data Mining Approach: What Determines the Wellbeing of Women in Montenegro, North Macedonia, and Serbia?

**DOI:** 10.3389/fpubh.2022.873845

**Published:** 2022-06-01

**Authors:** Vesna Bjegovic-Mikanovic, Helmut Wenzel, Ulrich Laaser

**Affiliations:** ^1^Faculty of Medicine, Belgrade, Serbia; ^2^Consultant, Konstanz, Germany; ^3^Faculty of Health Sciences, Bielefeld University, Bielefeld, Germany

**Keywords:** happiness, life satisfaction, subjective wellbeing, MICS, Montenegro, North Macedonia, Serbia, women

## Abstract

**Background:**

Women's happiness and life satisfaction, often summarized as subjective wellbeing, are of great value for most individuals and are associated with various determinants. The countries of the Western Balkan are of particular interest after the political changes in the nineties. Are the women satisfied with their lives today?

**Methods:**

We use the most recent datasets of the Multiple Indicator Cluster Surveys (MICS) for women 15–49 years old and with comparable data coverage for three countries of the Western Balkan belonging to the former Yugoslavia, namely Montenegro, North Macedonia, and Serbia. After sorting out variables of limited relevance or quality (missing values >50%), the remaining 32 variables followed a descriptive analysis. Four potential determinants of subjective wellbeing (SWB), an integration of happiness and satisfaction with life, entered an interactive Classification and Regression Tree (iC&RT) to account for their mostly bivariate format: age, education, region, and wealth.

**Results:**

The iC&RT analysis determines the influence of 4 independent variables (age, education, region, and wealth) on overall happiness, satisfaction with life, and subjective wellbeing, resulting in a high overall SWB of 88.9% for Montenegro, 82.1% for North Macedonia, and 83% for Serbia. The high relevance of younger age, higher education, and wealth, as critical determinants of a high SWB, and the lesser role of regions except for Serbia is confirmed. The spread of SWB in defined population subgroups ranges from 80.5–92.6% for Montenegro, 64.2–86.8% for North Macedonia, and 75.8–87.4% for Serbia.

**Conclusions:**

The three selected South-Eastern European countries of the former Yugoslavia (Montenegro, North Macedonia, Serbia) represent high levels of subjective wellbeing of women and a narrow range between the lowest and highest population groups. Women in Montenegro take a top position regarding their subjective wellbeing.

## Introduction

Personal happiness and life satisfaction are two terms of great value for most individuals. Still, they are difficult to define precisely. They stand for emotions and perceptions of life, which may refer to different circumstances and the impression upon a personality due to their upbringing, experience in life, and beliefs (1, 2). For these reasons, the scientific literature increased only recently (3), but still lacks uniformly accepted and precise definitions of what is meant (4). Nevertheless, modern science allows for the first time to quantify happiness, a subject of moral philosophies debated since Aristotle (5).

In the literature, various additional terms are discussed, the foremost is wellbeing, often divided into three subdomains capturing the experience of positive feelings (hedonic wellbeing corresponding to happiness), levels of satisfaction with life (evaluative wellbeing), and a sense of purpose and meaning (eudemonic wellbeing) (6). As indicated by several authors (7–9), subjective wellbeing (SWB) is a result of two primary factors: happiness and satisfaction with life, both with an impact on resilience (10). Many of the discussed determinants of SWB can be considered in the context of national or regional culture, defined by UNESCO (11) as “…the set of distinctive spiritual, material, intellectual and emotional features of society or a social group, and that it encompasses, in addition to art and literature, lifestyles, ways of living together, value systems, traditions, and beliefs.” Other studies confirm social cohesion and social capital as supportive of happiness [e.g., (12)]. The specific connection to education is discussed by Saevi (13), who describes the North American model as “psychological and managerial motivation oriented toward educational success,” whereas the “European pedagogy …had stronger structures of a rather contradictory human existential reflection.”

Evidence about the relationship between age and wellbeing is mixed. In the Western world, the connection is best explained by a U-shaped curve, the lowest levels of wellbeing in the middle age groups. Latin America shows a similar pattern, whereas, in sub-Saharan Africa, we see only minor changes over time (6). Respondents from the former Soviet Union and Eastern Europe, on the other hand, show a sizeable progressive decline in wellbeing with age [see also (14, 15)].

Gender usually is found to be a significant predictor of wellbeing. However, there is mixed evidence as to which gender experiences higher wellbeing. Combined multivariate analysis of the 2009 and 2011 *Scottish Health Survey* data indicated that men had higher odds than women for positive wellbeing. An analysis of the Annual Population Survey 2011-2012 data found that women had a higher overall wellbeing (16). Moreover, it has been reported that hedonic wellbeing was higher in men and eudemonic wellbeing higher in women (17, 18). Regarding gender issues, the Longitudinal Study of Young People in England interviewed a cohort of respondents annually from 2004 (then at age 13) until 2010. Data from 2010 indicated that at age 19, young people who identified as heterosexual “were more likely to be satisfied with their life” than those who identified as homosexual or bisexual (19).

Furthermore, a mutual influence between the dimensions of happiness and health has been confirmed repeatedly (3, 6, 17). In the prospective United Kingdom Million Women Study (20), happiness did not relate to mortality, while Kim et al. (21) and Trudel-Fitzgerald (22) report associations with cause-specific mortality. In addition, a unique, amenable living environment (23–25) can positively influence happiness and related parameters, although it is connected to wealth and health.

According to Inglehart and Klingemann (26) and Ye et al. (8), the differences in wellbeing vary relatively little over time within a country or region (vertical temporality), but between countries, it can vary even by one to ten (horizontal temporality). The relative vertical stability is connected to people's adjustment if they repeatedly experience negative affect. They become less demanding (27–29).

Since the end of the last century, the countries in South-Eastern Europe (SEE) have stabilized, some in connection with their membership in the European Union (Croatia, Slovenia) and others in a protracted accession process like Bosnia-Herzegovina, Montenegro, North Macedonia, and Serbia, all with related Slavic languages and therefore cultural commonalities, and the bilingual territory of Kosovo^1^ with Albanian and Serbian language. In addition, all of them except Albania belonged to the Yugoslavian state, established after World War I in 1918/19, broken apart in the civil wars of the 1990's. Except for Kosovo, all are characterized by the dominant Christian—catholic or orthodox —religion. Women have been confined to traditional roles for centuries under the long-lasting Ottoman rule^2^ in these countries (30), likely to be less individualistic and more collectivistic (31) than in neighboring central and Western Europe. The recent generations of women enjoyed increasingly equal acceptance, especially during the socialist period under Tito (1892-1980), and are now requesting their place in the modernizing South Eastern societies (32). Several recent articles, theoretical approaches, and studies address determinants of subjective wellbeing of the general population or vulnerable groups in the European and SEE regions. However, women are rarely targeted as the sole population.

This study explores determinants of women's subjective wellbeing in three selected countries of South-Eastern Europe: Montenegro, North Macedonia, and Serbia. We hypothesize that several factors influence different groups of women's subjective wellbeing. Furthermore, we expect to observe differences between selected countries, highlighting horizontal temporality despite a joint historical development.

## Methods

In a cross-sectional approach, we analyze the latest MICS surveys available from the UNICEF database (33) of women 15–49 years old and implemented between 2018 and 2019 in three South-Eastern European (SEE) countries (see Box 1) with related Slavic languages, history, and culture, i.e., Montenegro, North Macedonia, and Serbia, which comprise 50.4% of the former Yugoslavia's population. Bosnia-Herzegovina (BiH) and Kosovo could not be integrated, as for BiH the survey data from 2020 are not yet available, and for Kosovo, the dependent variables of happiness and life satisfaction, unfortunately, have not been reported in the latest survey. Regarding the two remaining Yugoslavian succession states, the last MICS in Croatia dates more than 20 years back. For Slovenia, a survey has never been run. Repeatedly, we draw on experience presented in our first publication about women's happiness in Montenegro (7). Therefore, we use Montenegro as a reference country where required. Furthermore, we investigate whether the variable groups related to subjective happiness, i.e., those describing grief and threats and those relating to health services during pregnancy show differences between analyzed countries.

Box 1 **Latest MICS surveys.**

**Table:** 

Bosnia-Herzegovina	(2020?)
Croatia	1996
Kosovo	2020
**Montenegro**	**2018**
**North Macedonia**	**2019**
**Serbia**	**2019**
Slovenia	-.-

Participation has been determined as follows, taking the reference example of Montenegro (34): of the 6,000 households selected for the national sample, 5,416 were found occupied; 3,826 households were successfully interviewed, corresponding to a household response rate of 70.6%; and 2,928 women (age 15–49 years) were identified in the interviewed households, corresponding to 76.5%. Of these, 2,276 were successfully interviewed, yielding a response rate of 77.7%. In the case that all 5,416 households of the sample were successfully interviewed, not 2,928 but ~4,145 women in the interviewed households could have been identified (given the same percentage of women per household i.e., 76.5%). The 2,276 interviewed women make up only 54.9% of the representative sample related to this potential sample size of 4,145 women. It remains an open question whether self-selective dynamics played a role here.

The MICS datasets^3^,two-stage stratified cluster samples, for the three available South-Eastern European countries contain, for e.g., for our standard reference Montenegro, 383 variables (out of which 27 relate to organizational procedures and 4 to the dependent variables of happiness and life satisfaction). This leaves in the example of Montenegro 352 independent variables with a potential impact for the analysis, almost all categorical. The basic questionnaire can be found in the UNICEF database (33), nearly identical for all three countries (where not, this is mentioned in the tables). To reduce the number of variables and select a manageable set of potential predictors, we applied a module for “Feature selection and variable screening” (35) as a pre-processor for predictive data mining. In a second step, we checked all remaining variables whether they have a response rate >50% in the sample of Montenegro. Otherwise, we did not make further use of them. Due to its relevance, we allowed for one exception: the year of first birth (CM16BY). For relevant groups with more than six variables, we kept only three to four variables providing the best spread and showing a prevalence of positive answers of ≥1.0% in Montenegro. This approach to reducing complexity concerned the variable group “Heard of contraceptive methods” (CP0A-N) and “Current use of contraceptive methods” (CP4A-N) further down in **Table 4**. Finally, in a third step, we eliminated all variables not available in all three countries subjected to our analysis (with one exception in **Table 5** further down and one in **Table 6** regarding the descriptive presentations there). For WB15 (“Duration of living in current place”), we replaced the answer “Always/since birth” with the age of the woman.

The final list of potential determinants and their rates of missing values is shown in Table 1, together with the four dependent variables of happiness and life satisfaction. Furthermore, in Table 2, we categorized the remaining variables according to three themes: I. medical assistance; II. grief and threats; III. marriage and children. A preceding category assembles the four available discriminators A–D: age of women, education, region, and wealth. We did not use weighted averages between the three selected countries; we wanted to analyze the “real” situation independent of differing determinants.

**Table 1:** Selection of variables with missing values.

**Sections**	**Line number of related areas**	**Selection of variables/line numbers based on missing values <50% of the Montenegrin sample (*N* = 2928)**	**Variable Acronym**	**Montenegro** Missing values^*^ ***N* = 2928**	North Macedonia Missing values*: *N* = 3169	**Serbia** Missing values*: *N* = 4219
**Discriminating variables A-D**						
A. Age	30	30-Age of women	WB4	22.3	6.5	11.4
B. Education	31, 32, 33–41, 361	361-Education	welevel	22.3	6.5	11.4
C. Region	42–44, 46–51, 369	42-Duration living in the current place (364) 369-Region	WB15 HH7	22.3 0.0	6.5 0.0	11.4 0.9
D. Wealth	372–380	373-Wealth Index Quintile	Windex 5	22.3	6.5	11.4
**Selected themes** **I-III**						
I. Medical assistance	87–174, 276–283,	88-Prenatal care provider: Doctor 89- Prenatal care provider: nurse/midwife^**^	MN3A MN3B	0.0 0.0	0.0 0.0	0.0 0.0
	306–320	101-Assistance at delivery: Doctor 102-Assistance at delivery: nurse/midwife^**^	MN19A MN19B	0.0 0.0	0.0 0.0	0.0 0.0
II. Grief & threats	52–54, 232–267	52-Ever had a child who later died (357) 232-236 Beating by husband 261-266 Felt discriminated	CM8 DV1A-E VT22A-F	22.3 22.3 22.3	6.5 6.5 6.5	11.4 11.4 11.4
III. Marriage & children	45, 46, 55–86, 175–231, 268–275, 284–305, 353, 355	46-Any sons or daughters living with you 58-Year of last birth^***^ 60-Year of first birth^***^ 62-Life births in last two years 175–187 Heard of contraceptive methods 191-Ever used method to avoid pregnancy 192-204 Current contraceptive methods 229-Availability of private place for washing during last menstrual period	CM2 CM15Y CM16BY CM17 CP0A-N CP3 CP4A-N UN17	45.3 42.3 54.0 45.3 22.3 34.4 22.3 26.3	30.0 1.2 0.5 30.0 6.5 54.0 0.0 0.0	11.4 34.8 34.8 34.8 11.4 58.3 0.0 0.0
		268-Currently married or living with a man 353-Age at first marriage/union of a woman	MA1 WAGEM	22.3 22.3	6.5 25.8	11.4 30.7
		355-Children ever born	CEB	42.7	6.5	11.4
**Dependent variables**	345	Estimation of overall happiness	LS1	22.3	6.5	11.4
	346	Satisfaction with ladder step	LS2	22.3	6.5	11.4
	347	Life satisfaction in comparison with last year	LS3	22.3	6.5	11.4
	348	Life satisfaction expectation 1 year from now	LS4	22.3	6.5	11.4

*Variables are excluded if <50% of the total sample size of N=2928 in the reference Montenegro are available*.

* *% of sample size N*.

** *Assisting the physician or alone*.

*** *Alternative variable names: BH4_FIRST and BH4_LAST for North Macedonia and CM15AY for Serbia*.

**Table 2:** Women's descriptive characteristics and subjective wellbeing (SWB).

**Sections**	**Line numbers and variable names (abbreviated)**	**Acronyms**	**MN**	**NM**	**SRB**
	**Total sample, incl. missing values**		2928	3391	4378
**Standard discriminators**	**National values are given as valid % or as specified**				
A. Age of women	30-Age of women (average)	WB4	32.6	32.5	33.5
B. Education	361-Education: The highest level of the school attended (percent higher than secondary level)	welevel	32.6	33.8	41.1
C. Region	42-Living in current place since birth (percent) 369-Region (percent in the central region)	WB15 HH7	46.8 35.8	51.7 19.7	53.7 24.3
D. Wealth	373-Wealth Index Quintile (percent of quintiles 4 & 5)	Windex5	40.9	31.8	41.5
**Themes I – III**					
I. Medical assistance (All variables as a percentage)	88-Prenatal care provider: doctor 89- Prenatal care provider: nurse/midwife^*^ 101-Assistance at delivery: doctor 102-Assistance at delivery: nurse/midwife^*^	MN3A MN3B MN19A MN19B	98.6 11.3 93.7 89.6	98.1 5.4 96.7 92.7	99.2 19.1 93.8 92.3
II. Grief & threats (All variables as a percentage)	52-Ever had a child who later died (357) 232-If she goes out without telling her husband, wife-beating justified	CM8 DV1A	1.7 2.2	2.6 4.4	1.0 0.5
	233-If she neglects the children: wife-beating justified 234-If she argues with her husband: wife-beating justified 261-In the past 12 months, felt discriminated against ethnic or immigration origin	DV1B DV1C VT22A	4.3 1.6 1.4	9.4 4.6 3.5	1.4 0.7 1.5
	262-In the past 12 months, felt Discriminated against gender	VT22B	1.2	4.2	3.1
	263-In the past 12 months, felt discriminated against for religion or belief	VT22E	1.2	1.6	0.7
III. Marriage & children	46-Any sons or daughters living with you (percent) 58-Year of last birth (years ago, median) 60-Year of first birth (years ago, median) 62-Life deliveries in the previous 2 years (percent) Knowledge of birth-control methods as a percentage:	CM2 CM15(A)Y^**^ CM16BY^**^ CM17	98,1 4 12 26.9	98.2 6 11 24.2	97.5 4 9 24.0
	179-Heard of: implants 183-Heard of: diaphragm 186-Heard of: withdrawal 191-Ever used a method to avoid pregnancy 194-Current method: IUD 198-Current method: male condom	CP0E CP0I CP0M CP3 CP4C CP4G	28.6 53.4 83.6 12.0 2.0 3.8	39.4 51.8 93.2 23.6 1.0 10.1	46.9 79.9 97.9 37.0 1.4 14.4
	202-Current method: periodic abstinence/rhythm method	CP4L	1.0	1.6	8.3
	203-Current method: withdrawal 229-Availability of private place for washing during last menstruation (percent)	CP4M UN17 MA1	4.6 97.5 69.2	37.5 98.0 75.9	28.5 98.8 71.4
	268-Currently married or living with a man (percent) 353-Age at first marriage/union of women (median) 355-Children ever born (median)	WAGEM CEB	23 2	22 2	23 2
**SWB and composite components**	345-Estimation of overall happiness (1st of 5 levels = best), levels 1 and 2: very and somewhat happy (percent)	LS1	73.9	85.2	79.8
	346-Satisfaction with life according to ladder step (10^th^ of 10 levels = best), levels 7–10 (percent)	LS2	68.4	70.8	73.2
	347-Life satisfaction in comparison with last year (level 1 best of 3 levels, percent)	LS3	35.6	44.4	40.0
	348-Life satisfaction expectation 1 year from now (level 1 best of 3 levels, percent)	LS4	55.6	75.1	68.6
	Subjective Wellbeing: SWB = LS2 - (2.5 ^*^ LS1); range: −11.5–7.5 = best (mean); *N* = 2,204 (missing 24.7%)	SWB	5.35	4.05	4.39

* *Assisting the physician or alone*.

** *Alternative variable names: BH4_FIRST and BH4_LAST for North Macedonia and CM15AY for Serbia*.

*Reference is the total samples of N = 2.928 resp. 3169 resp. 4219 minus missing values (see Table 1)*.

*The variable groups CP0, CP3, and CP4 are represented here only by selecting a small number of typical items (8 out of 27)*.

*MN, Montenegro; NM, North Macedonia; SRB, Serbia*.

The various MICS provide several categorical (YES/NO) indicators for health care quality. We checked the professional assistance provided to the mother during pregnancy and delivery (Table 3 further below). As the total number of births in the last 2 years is unknown, the estimation was done based on whether women had one or more children during the previous 2 years (answer: YES/NO). However, it is unlikely that a more significant number had two or even three deliveries during this short period. For Serbia, postnatal services were unavailable, but we added the two variables, “Mother checked after delivery” and “Baby checked after delivery,” available for Montenegro and North Macedonia.

**Table 3:** Professional assistance during pregnancy.

	**Variable name**	**Montenegro**	**North Macedonia**	**Serbia**
**Variable abbreviation**	**Total number of at least one live birth in the last 2 years (CM17)**	432 (100%)	574 (100%)	660 (100%)
MN3A&B	**Prenatal care provider:**			
	doctor nurse/midwife (alone)	426 (98.6) 1 (0.2)	563 (98.1) 2 (0.3)	655 (99.2) 2 (0.3)
MN19A&B	**Assistance at delivery:**			
	doctor nurse /midwife (alone)	405 (93.7) 26 (6.0)	555 (96.7) 19 (3.3)	619 (93.8) 40 (6.1)
PN14A&B	**Baby checked after delivery:**			
	doctor nurse/midwife (alone)	295 (68.3) 21 (4.9)	258 (44.9) 281 (49.0)	n.a.
PN23A&B	**Mother checked after delivery:**			
	doctor nurse/midwife (alone)	177 (41.0) 4 (0.9)	284 (49.5) 35 (6.1)	n.a.

Deviating from our first analysis (7), we extended the concept of defining the dependent variable here, analogous to Inglehart et al. (36), who suggest that combining the variables of happiness and life satisfaction provides a broader-based and more reliable indicator of the subjective wellbeing (SWB) levels of societies than do either of its two components (for the involved variable names in the following formula see Tables 1 or 2 at the last group of variables). For this procedure, we use the formula proposed by them, where the dependent variable SWB = LS2 - (2.5 ^*^ LS1). The maximum value here is SWB = 10 – (2.5 ^*^ 1) = 7.5 and the minimum is SWB = 1 – (2.5 ^*^ 5) = −11.5 (the distance being 19 points), not counting missing values and zero values.

The MICS dataset is also analyzed by subnational regions, where available, to represent a potentially closer social relatedness. In addition, we use available parameters at the national level to link to each other all three social layers, i.e., the individual micro-, the regional meso-, and the national macro-level (34). At the national level, the following are available: population density, female life expectancy, gross domestic product (GDP), distribution of gender in the national workforce (job information is missing in MICS), human rights index, corruption index, trust level, human freedom index, charity index, and human development index (see **Table 7** further below).

In a first step, we performed a stepwise regression to determine the relationship between the remaining variables A-D and the SWB, despite the large scattering of the measured data. However, the coefficient of determination (adjusted R^2^) is low, i.e., 4.4% in the Montenegrin model, 5.97% in the Serbian model, and 9.9% in the North Macedonian model. At the same time, the *p*-values are highly significant. Concerning the question of which R-value is appropriate and sufficient, the general view in the literature is that values above 70% are desirable (35). Some authors point out that in cases where it is about the correlation of variables and not about predictions, the *p*-value may be more critical than the R^2^-value (36, 48). Even high-variability data can have a significant trend (48, 49). Nevertheless, we preferred to treat these results cautiously and performed further analyses using the data mining technique (50–52), more precisely, an interactive Classification and Regression Tree (iC&RT) (35). Unlike our first categorical C&RT analysis (7), we applied a C&RT regression here. This allowed us to present mean values for SWB and associated variances at all positions of the iC&RT trees.

a) The Interactive Trees module (iC&RT) allows the use of both categorical and interval scaled variables, is optimized for vast data sets, and is also more flexible in handling missing data^4^. The program runs predictors, one at a time, to determine the best (next) split of the starting population and the subsequent subgroups at lower levels. For example, in the General CHAID (GCHAID) module, observations with missing data for any categorical predictor are eliminated from the analysis, and variables with insufficient/lesser variance in comparison (53).

b) iC&RT allows “what-if” analyses by interactively deleting individual branches, growing other components, and observing different result statistics for the various trees (tree models).

c) One can automatically grow some tree parts, but manually specify splits for other branches or nodes to find and specify alternative predictors and partitions.

d) One can define specific splits to build economical and straightforward solutions that can easily be communicated and implemented.

e) Reloading, the tree will be restored to the same state as it was saved (54).

The advantage of high flexibility, on the other hand, requires answering the question of how to find the “right size” of a tree. A too high or low complexity can dilute the model's statement. A very complex tree provides many insights that might be overlooked in a more straightforward tree. It risks creating nodes filled with minimal numbers. So, it is up to the analyst to select meaningful trees. In this way, it compromises simplicity, accuracy, and meaningfulness. Therefore, we controlled for both aspects with cross-validation in the iC&RT model and verified this model with a cross-check of an iC&RT analysis. In the latter, the adequacy of the model solution was checked according to the one standard error rule (54–56). Both approaches provide compatible results.

## Results

After the selection process, described in the Methods section, to identify potential determinants of the dependent variables, Happiness and Life Satisfaction, the variables listed in Table 2 remained for the resulting analyses, with 29 potential determinants and four dependent variables together with two integrated indices. We added selected descriptive variables in Tables 3, 4 below for a complete picture (maternal care and birth control).

**Table 4:** Knowledge and use of selected birth control methods.

**Distribution by**	**Information or Practice**	**Montenegro**		**North Macedonia**		**Serbia**	
		**Spearman** **Rank r**	***p*-value equal to**	**Spearman** **Rank r**	***p*-value equal to**	**Spearman** **Rank r**	***p*-value equal to**
**Age**	**Ever heard of…**						
CP0E	Implants	−0.012	0.554	−0.070	0.000	0.010	0.553
CP0I	Diaphragm	−0.081	0.000	−0.041	0.000	−0.103	0.000
CP0M	Withdrawal	−0.161	0.000	−0.165	0.000	−0.113	0.000
**Education**	**Ever heard of…**						
CP0E	Implants	−0.180	0.000	−0.250	0.000	−0.118	0.000
CP0I	Diaphragm	−0.292	0.000	−0.355	0.000	−0.190	0.000
CP0M	Withdrawal	−0.154	0.000	−0.130	0.000	−0.094	0.000
	**Ever used…**						
CP3	Any contraceptive method	−0.112	0.000	−0.115	0.000	−0.161	0.000
**Wealth**	**Ever heard of…**						
CP0E	Implants	−0.168	0.000	−0.218	0.000	−0.085	0.000
CP0I	Diaphragm	−0.260	0.000	−0.321	0.000	−0.187	0.000
CP0M	Withdrawal	−0.168	0.000	−0.138	0.000	−0.074	0.000
	**Ever used…**						
CP3	Any contraceptive method	−0.136	0.000	−0.185	0.000	−0.175	0.000

Table 3 shows almost complete prenatal care coverage and delivery by a physician. In contrast, postnatal care of mother and child in the two available countries, Montenegro and North Macedonia, lacks appropriate coverage. A nurse or a midwife generally assists the physician in all functions with one exception: in North Macedonia, the nurse checks the baby after delivery independently in 49.0%. The data also illustrate women's subjective wellbeing through maternal care and birth control.

Furthermore, we analyzed in Table 4 knowledge (“ever heard of…”) and the use of birth control methods selected in Table 2. Contrary to the wealth index, age and education significantly impact knowledge and use of birth control methods except implants (did you ever hear of implants?) in Montenegro and Serbia, see Table 4.

We did not further explore section II in Table 2 on grief and threats. The low level of positive answers did not allow for more advanced analyses.

As described earlier, we identified four discriminators of subjective wellbeing (SWB): age of women (WB); education (welevel); wealth index quintile (WB5); region (HH); and duration of living in the current place (WB1new^5^). The spectrum of SWB ranges from−11.5 to +7.5, i.e., 19 units equal to 100%. Based on the iC&RT analysis, the nodes describe a defined population i.e., a group of similar women.

The results of the iC&RT analyses in Table 5 and the corresponding figures in Annexes I–IV demonstrate the distribution of subjective wellbeing (SWB) according to the four discriminators, subjecting the female population of the three chosen countries, based on the respective MICS survey data.

**Table 5:** Comparison of the end-nodes (defined women groups) of the three countries under consideration (for details, see the iC&RT figures in Annexes I–III, and the statistical evaluation in Annex IV).

**Montenegro**				**North Macedonia**				**Serbia**			
			**SWB** **Mean**				**SWB** **Mean**				**SWB** **Mean**
ID^*^	**N**	**%**	**±SD**	ID^*^	***N***	**%**	**±SD**	ID^*^	***N***	**%**	**SD**
01	2184	100	5.4 ± 2.5	01	2998	100	4.1 ± 3.3	01	3627	100	4.4 ± 2.9
43	247	11.3	6.1 ± 1.8	69	838	28.0	5.0 ± 2.6	29	1073	29.6	5.1 ± 2.4
42	614	28.1	5.6 ± 2.2	79	151	5.0	4.6 ± 2.7	26	154	4.3	4.6 ± 2.5
34	588	26.9	5.6 ± 2.2	66	327	10.9	4.3 ± 3.5	28	987	27.2	4.5 ± 2.6
28	202	9.2	5.1 ± 2.8	68	997	33.3	4.3 ± 2.7	39	620	17.1	4.4 ± 2.9
35	244	11.2	5.0 ± 2.3	78	342	11.4	3.1 ± 3.4	41	195	5.4	4.1 ± 2.8
31	103	4.7	4.6 ± 2.8	67	138	4.6	2.3 ± 4.5	27	180	5.0	3.4 ± 3.4
29	186	8.5	3.8 ± 3.5	64	90	3.0	2.0 ± 4.7	40	418	11.5	2.9 ± 3.7

* *Identification number of node respectively the identified subpopulation of women, see Annexes I–III*.

The SWB levels at the starting nodes in Table 5 differ only to a small extent (Cohen's D is between 0.07 and 0.43), nevertheless significant at *p* < 0.05%. Montenegro is ranked first with 88.9% of the maximum (full range from −11.5 to +7.5 = 19 points equalling 100%), followed by Serbia with 83.7%, and North Macedonia with 82.1%. Accordingly, the end-nodes indicate a relatively narrow spectrum of SWB in the three populations between the highest and lowest group in each: between 80.5 and 92.6% for Montenegro, 64.2 and 86.8% for North Macedonia, and 75.8 and 87.4% for Serbia. The spread between the least and the most wellbeing women group (comparing end-nodes) is the narrowest in Serbia (11.6%), the second in Montenegro (12.1%), and the highest in Macedonia (22.6%).

In Table 6, we add for Montenegro, considered the reference country (7), a detailed description of the iC&RT end nodes (finally determined population groups). The first three nodes (34, 53, 54) with the highest SWB means (between 5.6 and 6.1 of a maximum of 7.5) are characterized by a secondary or higher level of education, women of younger age (between 27 and 37 years), and, for node 43, living for several decades at the same place. As can be expected, they belong to the wealthiest population layers, L1 or L2. Still, there is also a group (between 52 and 56% in these three groups) that, despite secondary or higher education, belongs to the lower wealth levels (L3 or L4). The SWB level of these first three nodes, comprising two-thirds of the female population (66.3%), ranges between 5.6 and 6.1, corresponding to the 90th decile of the possible SWB spectrum.

**Table 6:** Detailed description of the terminal iC&RT nodes (defined women groups) of subjective wellbeing of Montenegrin women.

ID^*^	***N* = 2184**	**%**	**SWB Mean/SD**	**A** **Years** **mean age of** **women**	**B** **Education (%)** **Higher Secondary** **Primary or less**	**C1** **Years** **living in the same place**	**D** **Level of wealth (%) L1(richest)-L5**
43	247	11.3	6.1 ± 1.8	36.9	S (51.4)/H (46.6)	36.7	L1 (48.2) L4 (51.8)
42	614	28.1	5.6 ± 2.2	30.7	S (49.8)/H (47.4)	13.5	L1 (44.3) L4 (55.7)
34	588	26.9	5.6 ± 2.2	27.0	S (55.4)/H (36.7)	19.2	L2 (47.8) L3 (52.2)
28	202	9.2	5.1 ± 2.8	22.6	S (58.9)/P (28.7)	15.2	L5 (100.0)
35	244	11.2	5.0 ± 2.3	42.0	S (65.6)/P (18.9) H (15.6)	25.5	L2 (51.2) L3 (48.8)
31	103	4.7	4.6 ± 2.8	47.5	S (62.1)/H (21.4) P (16.5)	47.5	L1 (28.2) L2 (28.2) L3 (31.0) L4 (13.6)
29	186	8.5	3.8 ± 3.5	40.0	P (50.5)/S (44.1) H (5.4)	24.5	L5 (100.0)

* *Identification number of node respectively the identified subpopulation, see Annexes I-III*.

The remaining four nodes (28, 29, 31, 35) are characterized by smaller shares of highly educated women, but still a majority of women with secondary education except for the lowest group (node 29) with 50.5% of primary education only. This population comprises a majority of women in the higher age group (age 40–47.5) except for the 202 (5.1%) in node 28 with an average age of 22.6, obviously at the beginning of their professional career. Only 28.7% in this group and 100% of the women in node 29 belong to the lowest wealth level comprising 8.5% of the Montenegrin female population. This last group still reveals an average SWB level of 3.8, just in the 80th decile (80.5%).

North Macedonia (*N* = 2998) ranks in the top group at a node mean of 5.0 or 86.6% of the maximum SWB, comprising 28.0% of the female population 15–49 years old. The upper four nodes in Annex I represent 77.2% of the female population at SWBs between 4.3 and 5.0. The main splitting variables are the wealth index, age, and the Macedonian regions.

Serbia shows a somewhat higher top-level SWB of 5.1 or 87.4% for a group of *N* = 1,073 or 29.6% of the total (*N* = 3,627). Like in Montenegro and North Macedonia, wealth and age are the most critical splitting variables. However, the regions are highly relevant for Serbia, determining the end-nodes for 73.7% of the population.

Three-quarters of the difference in subjective wellbeing between the top 10 and bottom 10 countries and regions, according to the World Happiness Report of 2016 (57), can be explained by the following: (1) social support so that you have friends and family to count on, (2) freedom to choose what you do in life, (3) generosity and how much people donate to charity, 4) absence of corruption in business and government, (5) gross domestic product, and (6) healthy life expectancy.

At the national level, the most available relevant information for all seven Yugoslavian successor states is listed in Table 7. The indices selected suggest a superior position for Montenegro, except for the human rights index (Serbia ranked surprisingly 0.25 values higher in 2019) and the economic freedom index (North Macedonia ranked 0.40 higher). Sachs (57) summarizes the different views under six terms: mindfulness, consumerism, economic freedom, dignity of work, good governance, and social trust, modified further by Helliwell et al. (41) based on the results of Gallup surveys, including up to 157 countries (58, 59).

**Table 7:** National parameters of potential relevance, available for the six successor states of the former Yugoslavia compared to Austria.

**Country**	**Reference**	**Austria**	**Bosnia-Herzegovina**	**Croatia**	**Montenegro**	**North Macedonia**	**Serbia**	**Slovenia**
1) Population density/skm 2018	(37)	107	65	72	46	83	80	103
2) General and Female life expectancy, 2018 (years)	(38)	82.0 84.2	77.3 79.8	78.6 81.6	76.9 79.3	76.7 78.8	75.9 78.4	81.6 84.5
3) GDP 2019 (PPP$)	(39)	58.641	14.895	28.829	19.931	16.609	18.840	39.038
4) Share of women and men (%) employed in the labour force, 2019	(40)	55.9 67.1	32.8 51.1	45.2 57.6	49.9 62.2	44.9 66.1	47.1 62.7	53.2 63.0
5) Human Rights Index (1–10 best), 2019	(41, 42)	8.90	5.47	7.69	6.18	5.91	6.43	8.83
6) Corruption Index:	(43)							
Ranking, 2020 (1 = best) Score, 2020 (100 = best)		15 76	111 35	63 47	67 45	111 35	94 38	35 60
7) Trust Levels, 2008-2010 (100 = best)	(44)	36	27	20	25	19	12	24
8) Human Freedom:	(45)							
Ranking, 2019 (1 = best)		13/162	57/159	38/159	51/159	60/159	55/159	35/159
Index (1–10 best):								
Economic freedom Personal freedom		7.71 9.25	6.61 7.93	7.36 8.47	6.77 8.11	7.17 7.25	6.75 7.85	7.33 8.77
9) Charity, Ranking 2019 (1 = best)	(46)	15	90	132	116	130	129	47
10) Human Development Index, 2018 (0–1 best)	(47)	0.922	0.769	0.851	0.816	0.759	0.799	0.917

## Discussion

Modern strategies for health system development try to empower women and promote gender equality in governance and management at the macro (society and policy), meso (communities and institutions), and micro (social interaction in departments) levels. The WHO approach to gender mainstreaming (60) refers to projects and institutions striving to build capacities in developing gender equality, promoting sex-disaggregated data and gender analysis, and establishing accountability. Since women account on average for 70% of the workforce for health (61), gaps in health workers will decrease only by addressing the gender dynamics of the workforce. In its 5-year strategy (2019-2023), WHO (62) is committed to empowering countries for gender equity and a human rights approach in the day-to-day activities of the health sector. A recent review (63) pointed out that female health workers who deliver most of the care in all settings face barriers at work not faced by their male colleagues. This situation undermines their wellbeing and livelihood and constrains progress on gender equality. It negatively impacts health systems and the delivery of quality health services.

The three countries in this analysis represent about one-half of the former Yugoslavian population and still did not yet access the European Union. They have three more qualities in common: a former socialist constitution, the orthodox religion, and the long-lasting Ottoman rule. In addition, they rank relatively high, between the 42nd and 75^th^, positions in the World Happiness Index [(64), table 2.1], with an increasing tendency over the last years for all of them. We focus on young and middle-aged women under the original assumption that the cited history culturally determines them. However, we found the women in these three countries predominantly happy, measured by the more stable index of subjective wellbeing (SWB), which integrates overall happiness with life satisfaction. In addition, the women's outlook for the next year is very positive, increasing by 20% for Montenegro, 31% for North Macedonia, and 29% for Serbia.

Montenegro takes the top position underlined in our comparative analysis by comparing national indices. An explanation of Montenegro's unique position concerning its women is possibly a higher GDP, a higher share of the female workforce, a lower corruption index and score ranking, a better charity ranking, a better human development index, and accordingly, a higher life expectancy for both genders. The only exception is the human rights index of Montenegro, with a value of 6.18 in 2020 (in 2015: 6.92), whereas Serbia in 2020 takes the top position with 6.43 out of 10 points. In an earlier detailed analysis focused exclusively on Montenegro (7), we found for the two upper categories, very happy and happy (out of 5), a similar percentage of 96.7. However, to close up to Austria (first column in Table 7), a non-Yugoslavian country historically most related, will still need more years.

The generally high level of SWB and related parameters may also result from the high level of medical care expressed by the dominating physician's role referring to the example of medical care during pregnancy: health and happiness are mutually related (31, 65, 66).

The level of wealth is the most critical splitting variable in Montenegro, defining groups of similar SWB. In contrast, the regions play a dominant role in the two larger territories of North Macedonia and especially Serbia, in addition to age. However, human development models suggest that the emphasis shifts from the pursuit of happiness through economic means toward a broader perception by maximizing free choice in all realms of life, an option to increase perceived SWB (67, 68). The belief that one has free will and control over one's life is closely linked with happiness (69), and this link seems universal. Simovic (70) argues that Montenegro's extraordinary situation is due to the basic principles enshrined in the Montenegrin constitution, which are developed by a series of laws governing the exercise of the right to work, right to education, family relations, health, and social care.

To speed up interventions for gender equality, in 2017, WHO established the Gender Equity Hub (GEH), co-chaired by WHO and Women in Global Health under the umbrella of the Global Health Workforce Network. The GEH brings together key stakeholders to strengthen gender-transformative policy guidance and the implementation capacity for overcoming gender biases and inequalities in the health workforce, supporting the implementation of the Global Strategy on Human Resources for Health: Workforce 2030 (63). Gender analysis, empowerment, and mainstreaming became significant cross-cutting issues in developing capacity for health system management. There is much evidence confirming that the lack of gender parity in higher-level decision-making positions and leadership in the health workforce can influence the efficiency and quality of health services. In contrast, discrimination in health service settings can compromise Universal Health Coverage (64).

Our analysis has some limitations admittedly due to the quality of the sampling scheme and the considerable percentage of missing data. In the methods section, we pointed to the weaknesses of the sampling procedure as the potential sample size for women, e.g., for Montenegro aged 15–49, *N* = 3,826, leaving us with a participation rate of 54.9%. Furthermore, the low rates of positive answers in section II of Table 2 related to grief and threats did not allow more detailed analysis. The high rates of missing responses in section III of Table 2 on “Marriage & children” may have invalidated some of our results. Unfortunately, two indicators of high relevance in our context are missing in the MICS database: information on occupation/employment and social support. For these deficits, we chose a stable data mining approach.

## Conclusions

The three selected South-Eastern European countries of the former Yugoslavia (Montenegro, North Macedonia, and Serbia) present high levels of subjective wellbeing and a narrow range between the lowest and highest female population groups. Women in Montenegro take a top position regarding their subjective wellbeing.

## Data Availability Statement

Publicly available datasets were analyzed in this study. This data can be found here: https://mics.unicef.org/surveys.

## Author Contributions

All authors listed have made a substantial, direct, and intellectual contribution to the work and approved it for publication.

## Conflict of Interest

The authors declare that the research was conducted in the absence of any commercial or financial relationships that could be construed as a potential conflictof interest.

## Publisher's Note

All claims expressed in this article are solely those of the authors and do not necessarily represent those of their affiliated organizations, or those of the publisher, the editors and the reviewers. Any product that may be evaluated in this article, or claim that may be made by its manufacturer, is not guaranteed or endorsed by the publisher.

## Footnotes

^1^All references to Kosovo in this article should be understood to be in the context of the United Nations Security Council Resolution 1244/99 http://www.un.org/docs/scres/1999/sc99.htm.

^2^The Ottoman rule lasted from the battle of Kosovo in 1389 until the Berlin Congress in 1878.

^3^Permission obtained from UNICEF/MICS team by mail of 24.04.2020. This is a secondary analysis of the UNICEF surveys for which the authors obtained access and approval by UNICEF as the owner of the data.

^4^*Note: The iC&RT methodology uses the term “Node” to describe resulting subpopulations*.

^5^The answer “Always/since birth” has been replaced by the age of the women, resulting in 20 missing values.
