# Erratum: Data mining approach: What determines the wellbeing of women in Montenegro, North Macedonia, and Serbia?

**DOI:** 10.3389/fpubh.2022.1040582

**Published:** 2022-11-10

**Authors:** Frontiers Production Office

**Affiliations:** Frontiers Media SA, Lausanne, Switzerland

**Keywords:** happiness, life satisfaction, subjective wellbeing, MICS, Montenegro, North Macedonia, Serbia, women

Due to a production error, tables 1–6 were published incorrectly in the original version.

Table 1 on missing values was incorrectly published as Table 4.

Table 2 on data description was incorrectly published as Table 6.

Table 3 on obstetric assistance was incorrectly published as Table 5.

Table 4 on birth control methods was incorrectly published as Table 1.

Table 5 on C&RT endnodes was incorrectly published as Table 2

Table 6 on C&RT endnodes for Montenegro was incorrectly published as Table 3.

The publisher apologizes for this mistake. The original version of this article has been updated.

