# Erratum: Data mining approach: What determines the wellbeing of women in Montenegro, North Macedonia, and Serbia?

**DOI:** 10.3389/fpubh.2023.1147196

**Published:** 2023-02-09

**Authors:** Frontiers Production Office

**Affiliations:** Frontiers Media SA, Lausanne, Vaud, Switzerland

**Keywords:** happiness, life satisfaction, subjective wellbeing, MICS, Montenegro, North Macedonia, Serbia, women

Due to a production error, in-text citations on pages 3, 7, and 10 were entered incorrectly.

On page 3, right-hand column, paragraph 1, the citation was written as “Table 1.” It should be “Table 4.”

On page 3, right-hand column, paragraph 1, the citation was written as “Table 2.” It should be “Table 5.”

On page 3, right-hand column, paragraph 1, the citation was written as “Table 3.” It should be “Table 6.”

On page 3, right-hand column, paragraph 2, the citation was written as “Table 4.” It should be “Table 1.”

On page 3, right-hand column, paragraph 4, the citation was written as “Table 4 or 6.” It should be “Tables 1 or 2.”

On page 7, left-hand column, results section paragraph 1, the citation was written as “Table 6.” It should be “Table 2.”

On page 7, left-hand column, results section paragraph 1, the citation was written as “Tables 1, 5.” It should be “Tables 4, 3.”

On page 7, left-hand column, results section paragraph 2, the citation was written as “Table 5.” It should be “Table 3.”

On page 7, left-hand column, results section paragraph 3, the citation was written as “Table 1.” It should be “Table 4.”

On page 7, left-hand column, results section paragraph 3, the citation was written as “Table 6.” It should be “Table 2.”

On page 7, right-hand column, results section paragraph 3, the citation was written as “Table 1.” It should be “Table 4.”

On page 7, right-hand column, results section paragraph 4, the citation was written as “Table 6.” It should be “Table 2.”

On page 7, right-hand column, results section paragraph 6, the citation was written as “Table 2.” It should be “Table 5.”

On page 7, right-hand column, results section paragraph 8, the citation was written as “Table 3.” It should be “Table 6.”

On page 10, left-hand column, paragraph 2, the citations were written as “Table 6.” They should be written as “Table 2.”

The publisher apologizes for this mistake. The original files have been updated.

