# Supplementary material for: Data Mining Approach: What Determines the Wellbeing of Women in Montenegro, North Macedonia, and Serbia?
Source: Front Public Health. 2022 Jun 1;10:873845. doi: 10.3389/fpubh.2022.873845 (PMC9199491; doi:10.3389/fpubh.2022.873845)
Supplement: Supplementary file 1 [file Table_1.DOCX]

**ANNEX I**

iC&RT MONTENEGRO

*Note: Node 1 includes 20 missing values of the splitting variable WB15new. Therefore the corrected total N = 2184. The distribution of regionality (C2) is inconclusive.*

**ANNEX II**

iC&RT NORTH MACEDONIA

**ANNEX III**

iC&RT SERBIA

*(1) = BELGRADE, SOUTHERN AND EASTERN SERBIA*

*(2) = VOJVODINA, SUMADIJA AND WESTERN SERBIA*

*(3) = BELGRADE,VOJVODINA,SOUTHERN AND EASTERN SERBIA*

*(4) = SUMADIJA AND WESTERN SERBIA*

**ANNEX IV**

Significance and effect size (Cohen's D) of the overall SWB in Montenegro, North Macedonia, and Serbia (calculated online with special consideration to nonparametric tests (Mann-Withney U-Test) according to Lenhard (72).

| **Comparison of two countries** | | **Mann-Whitney**  **U test** | **Significance** | **Cohen’s D** |
| --- | --- | --- | --- | --- |
| Montenegro | N. Macedonia | 3127381 | p <.05 | 0.426 |
| Montenegro | Serbia | 2499436 | p <.05 | 0.372 |
| N. Macedonia | Serbia | 5216269 | p <.05 | 0.069 |
